# Supplementary material for: The lifetime of charged dust in the atmosphere
Source: PNAS Nexus. 2022 Oct 14;1(5):pgac220. doi: 10.1093/pnasnexus/pgac220 (PMC9802237; doi:10.1093/pnasnexus/pgac220)
Supplement: pgac220_Supplemental_Files [file pgac220_supplemental_files.zip › PNASNEXUS-PNASNEXUS-2022-00636-T-s01.pdf]

Supplementary Information for

The lifetime of charged dust in the atmosphere

Joshua Méndez Harper, Dana Harvey, Tianshu Huang, Jake McGrath III, David Meer, and Justin C. Burton

Joshua Méndez Harper.
E-mail: jmendez7@uoregon.edu

This PDF file includes:

Supplementary text
Figs. S1 to S4 (not allowed for Brief Reports)
Legend for Movie S1
SI References

Other supplementary materials for this manuscript include the following:

Movie S1

Supporting Information Text

Schlieren Imaging. The Tiny-lev acoustic levitator has a well defined theoretical acoustic field that has been explored in detail through simulations (1). To experimentally confirm our pressure field matched the theory, we imaged the time-averaged acoustic field using a single mirror off-axis schlieren system (Fig. S1) (2, 3). In our setup, a point source of light was created by placing an adjustable iris infront of a 632 nm LED light. The iris was adjusted to have a diameter of approximately 1 mm. The light traveled from the iris and passed through the acoustic field, then reflected off the front surface of a spherical mirror. The reflecting, front surface of the mirror was aluminized. The quality of the mirror was crucial; spherical aberrations in our previous attempts produced a broad focus unsuitable for imaging. After reflecting, the light was focused to a point of approximately the same size as the iris, where a razor blade is placed. The blade cuts off approximately half of the light from entering the camera lens. The final image consists of grey areas where the air pressure is ≈ 1 atm and black or white areas in areas of high and low pressure. The grey scale is associated with a magnification of the index of refraction due to changes in pressure and therefore density and index of refraction.

In order to maximize the visual differences between high and low pressure, the 40 kHz square wave that drove the transducers was synced to a high-speed camera (Phantom V7.11, Vision Research). The signal was clock divided to reduce redundant frames, so that movies were taken at 256 fps, and the exposure time was set to half the original period, 12.5 μs. This allowed the camera to capture only positive or negative pressure swings of the standing acoustic wave. To only capture a positive or negative pressure swing, we adjusted the delay between the beginning of a frame, and the beginning of the camera exposure in that frame. A median pixel combination of a sequence of images was applied to minimize ambient ambient air currents that our schlieren set up was sensitive enough to register. The final result is shown in Fig. 1 of the main text, where approximately 500 frames taken over 3 s were used. Similarly, we can view every image as the frame of a video to observe the acoustic field during a charge measurement (**Movie S1**)

Atmospheric Settling Model. To demonstrate the effect of decaying charge on the residence time of airborne particles, we performed a set of numerical experiments using a one-dimensional settling model. The dynamics of a spherical particle with density ρ_p and diameter D falling through an air column can be described by:

$$\frac{1}{6}\pi\rho_p D^3 \dot{v} = F_g + F_d + F_e, \tag{S1}$$

where v is the particle's velocity in the y-direction, and the dot refers to differentiation with respect to time. F_g, F_e, and F_d are the gravitational, electrostatic, and drag forces, respectively:

$$F_g = -\frac{1}{6}\pi D^3 \rho_p g, \tag{S2}$$

$$F_e = \pi D^2 \sigma(t) E, \tag{S3}$$

$$F_d = -\frac{1}{8} C_d \rho_a \pi D^2 v|v|. \tag{S4}$$

Above, g is the acceleration due to gravity, $\sigma(t)$ is the time-dependent surface charge density on the particle, and E is an ambient electric field. Note that the electrostatic force can either accelerate or retard sedimentation depending on the direction of the field and the polarity of the particle's charge.

Finally, ρ_a is the density of air, which depends on the altitude through the ideal gas law. For simplicity, we assume temperature varies with altitude according to a linear lapse rate $L = 0.0065$ K/m (4):

$$T = T_o - Ly. \tag{S5}$$

Above, $T_o = 273$ K is the temperature at sea level. Additionally, the variation of pressure with altitude p may be expressed as:

$$p = p_o (T/T_o)^{-(gM/(RL))}, \tag{S6}$$

where $p_o = 101$ kPa, $M = 0.02896444$ kg/mol is the molar mass of dry air, $R = 8.31447$ J K^{-1} mol^{-1} is the universal gas constant. From these relationships, the density of air may be computed as:

$$\rho_a = pM/(RT) \tag{S7}$$

This variation in rendered in **Fig. S3**. The drag coefficient C_d in Eq. S4 is computed using the formulation presented by Parmar et al. (5):

$$C_d = \frac{24}{\mathrm{Re}}(1 + 0.15\mathrm{Re}^{0.687}) + 0.42\left(1 + \frac{42500}{\mathrm{Re}^{1.16}}\right)^{-1}. \tag{S8}$$

Re is the Reynolds number given by $\rho_a|v|D/\mu$, where μ is the dynamic viscosity of air. **Equations 1** through **8** are solved numerically using the methodology described in Mastin et al. (6).

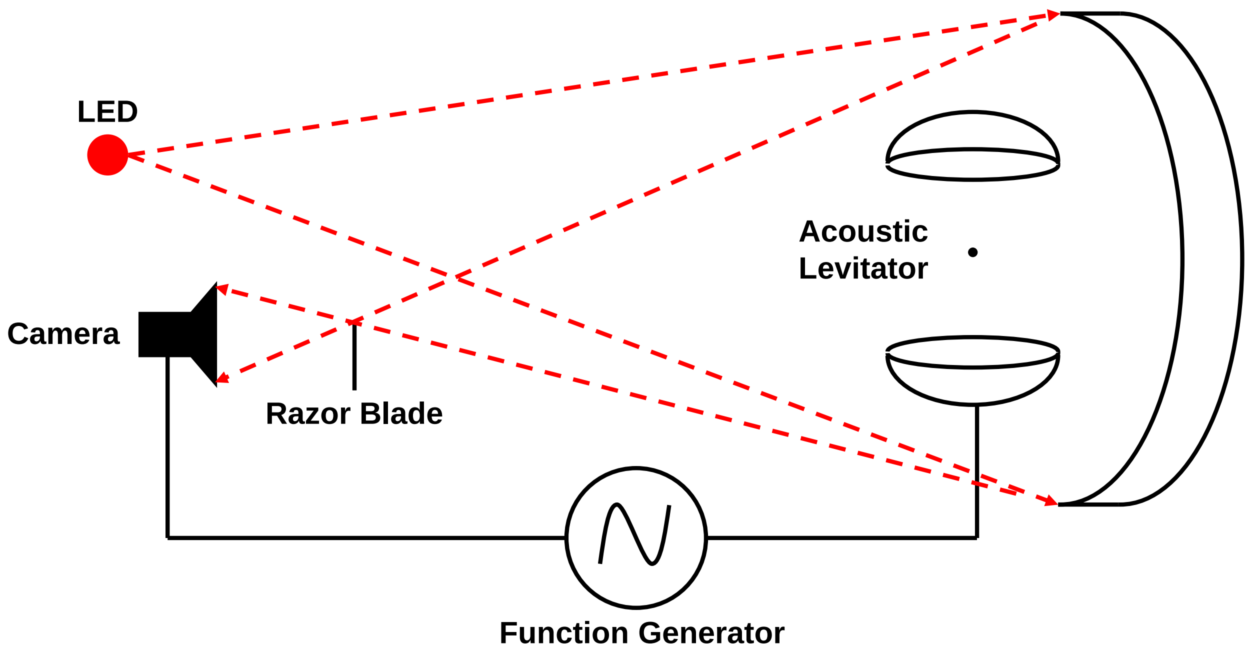

Fig. S1. The design of the schlieren imaging system. A monochromatic LED emits coherent light from a pinhole source, which hits a concave mirror of diameter 203 mm and focal length 750 mm. The mirror is placed 1.2 m away from the source. The acoustic levitator is placed adjacent to the mirror. The synchronized high-speed camera captures the light that passes above the razor blade. See reference (2) for additional details.

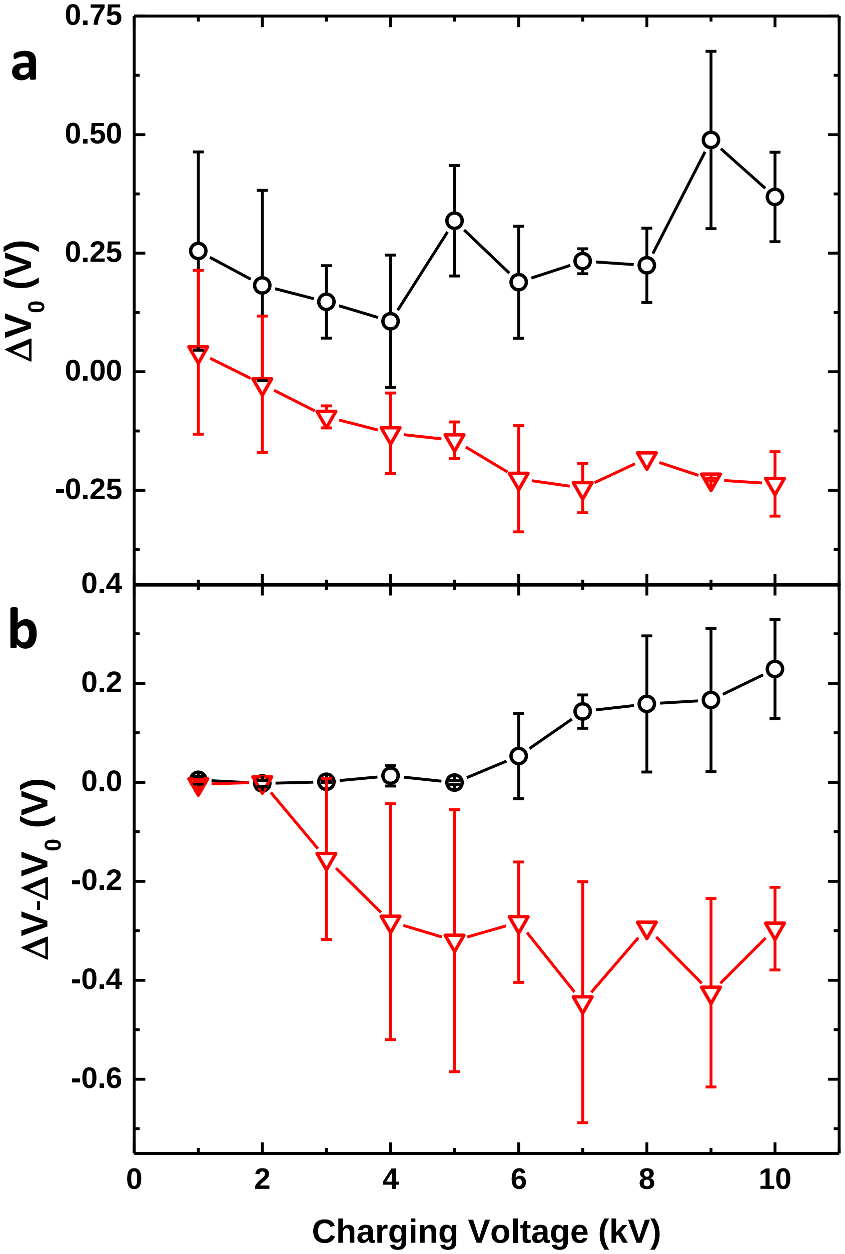

Fig. S2. (a) Average voltage obtained by a particle after charging with the corona ionizer. The ionizer is driven by a high-voltage power supply at a given voltage, as shown on the x-axis. Most particles have a natural positive charge before the experiment (Fig. 2 in the main text). (b) Average voltage difference between the final and initial state of a particle after charging with the corona ionizer. Charging mostly saturates after 7 kV. Our experiments typically use 8-10 kV.

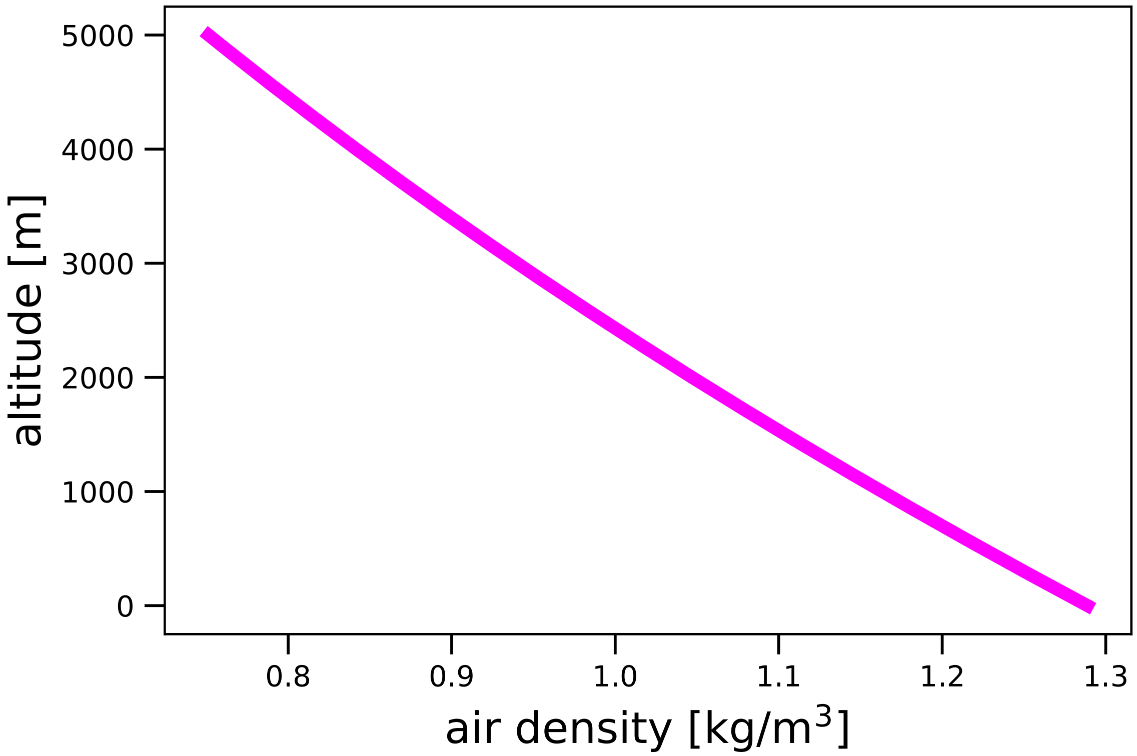

Fig. S3. Variation of air density with altitude used in this work. The model assumes a lapse rate of L=0.0065 K/m

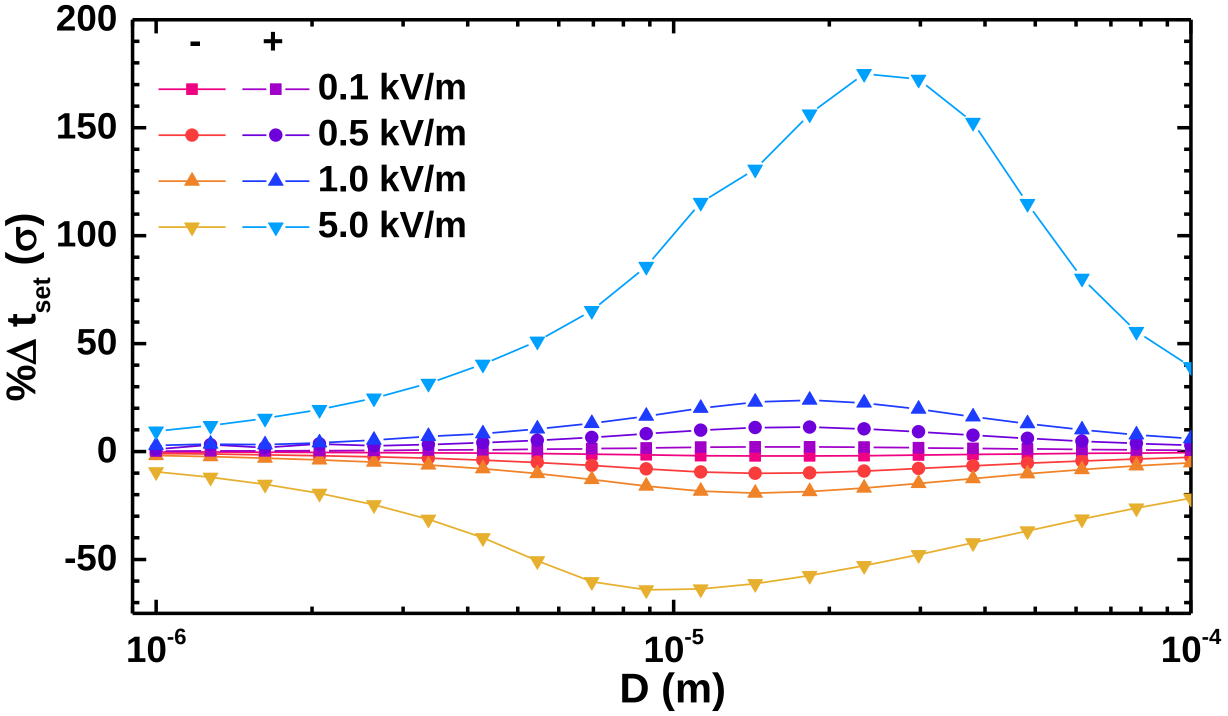

Fig. S4. Percent change in settling time for a positively-charged plastic particle (ρ = 1000 kg/m^3) in an external electric field with a surface charge density $\sigma = 10^{-5}$ C/m^2 that decay exponentially with $t_{1/2}$ = 4 days. The symbols represent different ambient electric fields ($+$, upward pointing; $-$ downward pointing).

 Joshua Méndez Harper, Dana Harvey, Tianshu Huang, Jake McGrath III, David Meer, and Justin C. Burton

Movie S1. Movie of a particle being manipulated to rise and fall within the levitator, viewed through schlieren imaging system. 1 mm = 27 pixels, and 1 s of video corresponds to 0.528 seconds of real time. The waves in the levitator change both in position but also in magnitude, as the particle rises and falls. This is a result of our capture method; an Arduino modifies the frequency of one of the transducer arrays in order to move the particle. This results in different phases of the pressure field to be captured during the exposure of the camera, leading to variations in pixel intensity as the particle moves.

References

1. A Marzo, A Barnes, B Drinkwater, Tinylev: A multi-emitter single-axis acoustic levitator. *Rev. Sci. Instrum.* **88** (2017).
2. A Crockett, W Rueckner, Visualizing sound waves with schlieren optics. *Am. J. Phys.* **86**, 870 (2018).
3. A Settles, GS, R Covert, EE, Schlieren and Shadowgraph Techniques: Visualizing Phenomena in Transport Media. *Appl. Mech. Rev.* **55**, B76–B77 (2002).
4. LG Mastin, A user-friendly one-dimensional model for wet volcanic plumes. *Geochem. Geophys. Geosystems* **8** (2007).
5. M Parmar, A Haselbacher, S Balachandar, Improved drag correlation for spheres and application to shock-tube experiments. *AIAA J.* **48**, 1273–1276 (2010).
6. LG Mastin, A simple calculator of ballistic trajectories for blocks ejected during volcanic eruptions. *U.S. Geol. Surv. Open-File Rep. 01-45* (2001).
